# Supplementary material for: Splice-Junction-Based Mapping of Alternative Isoforms in the Human Proteome
Source: Cell Rep. Author manuscript; Available in PMC 2020 Jan 15. (PMC6961840; doi:10.1016/j.celrep.2019.11.026)
Supplement: 2 [file NIHMS1546469-supplement-2.zip › DF1/PXD009737/TestisGluC/TestisGluC_26_NSD2_SKNPPTLSVQSVVKCIKMKQAPE.pdf]

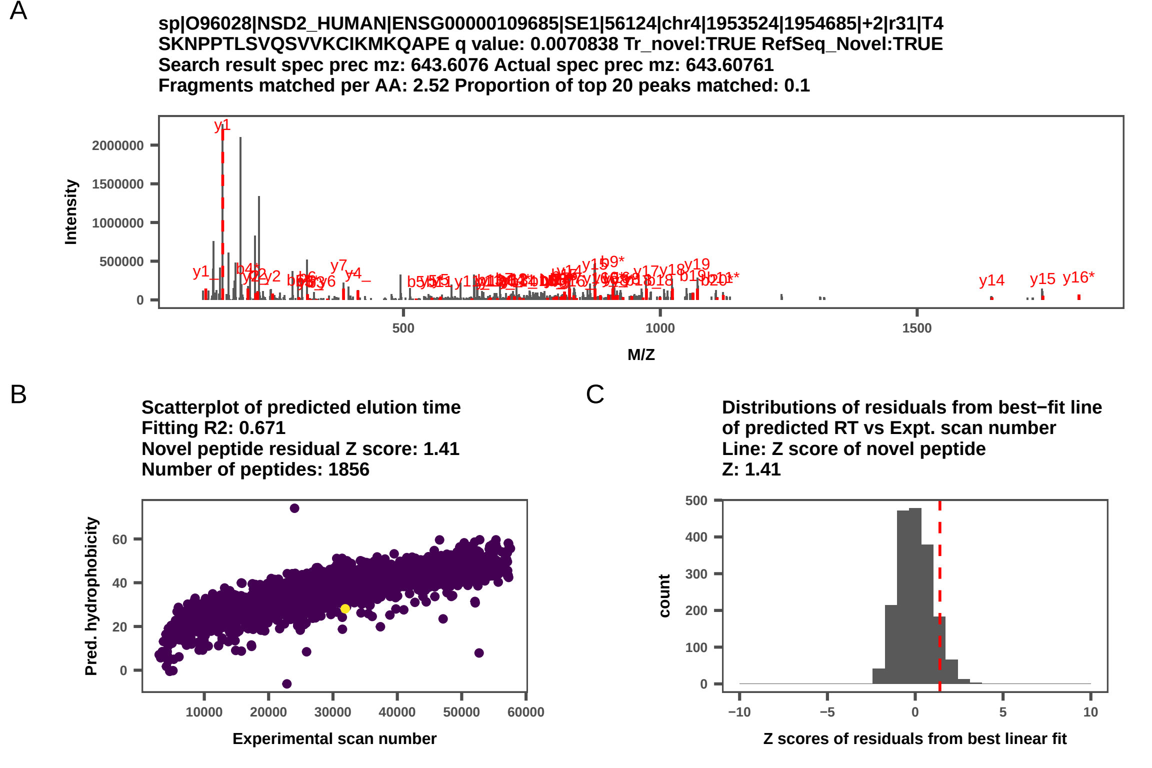
A
sp|O96028|NSD2_HUMAN|ENSG00000109685|SE1|56124|chr4|1953524|1954685|+2|r31|T4
SKNPPTLSVQSVVKCIKMKQAPE q value: 0.0070838 Tr_novel:TRUE RefSeq_Novel:TRUE
Search result spec prec mz: 643.6076 Actual spec prec mz: 643.60761
Fragments matched per AA: 2.52 Proportion of top 20 peaks matched: 0.1
Intensity
2000000
1500000
1000000
500000
0
500
1000
1500
M/Z
y1
y7
y14
y15
y16*
y19
B
Scatterplot of predicted elution time
Fitting R2: 0.671
Novel peptide residual Z score: 1.41
Number of peptides: 1856
Pred. hydrophobicity
60
40
20
0
10000
20000
30000
40000
50000
60000
Experimental scan number
C
Distributions of residuals from best−fit line
of predicted RT vs Expt. scan number
Line: Z score of novel peptide
Z: 1.41
count
500
400
300
200
100
0
−10
−5
0
5
10
Z scores of residuals from best linear fit
